# Capnography use in Scottish ICUs

**DOI:** 10.1186/cc10689

**Published:** 2012-03-20

**Authors:** C Wallace, S Cole, B McGuire

**Affiliations:** 1 Ninewells Hospital, Dundee, UK

## Introduction

Almost 20% of adverse airway events reported to the Royal College of Anaesthetists 4th National Audit Project (NAP4) occurred in the ICU [1]. NAP4 commented that the failure to use capnography probably contributed to 77% of the ICU airway mortality. NAP4 subsequently made a number of recommendations pertaining to capnography use. We designed a survey to describe practice with regards to these.

## Methods

A survey was sent to an intensivist at each of the 23 adult ICUs in Scotland.

## Results

There was a 100% response rate. Nineteen (83%) units used capnography for all tracheal intubations on the unit, two (9%) in over three-quarters, one (4%) in under one-half and one (4%) unit reported never using it. For tracheal intubations prior to unit admission, the corresponding usage was three (13%) always, seven (30%) in over three-quarters, seven (34%) in over one-half and six (26%) in less than one-half of all intubations. Continuous capnography monitoring was in use on 54% of the intubated patients and 63% of the ventilator-dependent patients. Twelve (52%) units reported using capnography in all the intubated and ventilated patients. Of the units not using continuous capnography routinely, two (18%) had no equipment for continuous monitoring.

## Conclusion

UK Intensive Care Society (ICS) guidelines make strong recommendations for the use of capnography in all critically ill patients during intubation [2]. We show a reassuring compliance with those guidelines during tracheal intubations performed on ICUs. Compliance was much poorer with the guidelines for those intubations performed outside units. An AAGBI safety statement recommended that continuous capnography should be used in all patients with intubated tracheas, regardless of location [3]. This was not echoed in the 2009 ICS guidelines (although in the light of NAP4, these have been updated to support this). Despite the majority of units in Scotland having facilities to monitor patients using capnography, just over one-half were doing so routinely. Capnography monitoring will surely increase in the advent of NAP4 and because of the change to the ICS guidelines.
